# Chitosan Coated Microparticles Enhance Simvastatin Colon Targeting and Pro-Apoptotic Activity

**DOI:** 10.3390/md18040226

**Published:** 2020-04-24

**Authors:** Nabil A. Alhakamy, Usama A. Fahmy, Osama A. A. Ahmed, Giuseppe Caruso, Filippo Caraci, Hani Z. Asfour, Muhammed A. Bakhrebah, Mohammad N. Alomary, Wesam H. Abdulaal, Solomon Z. Okbazghi, Ashraf B. Abdel-Naim, Basma G. Eid, Hibah M. Aldawsari, Mallesh Kurakula, Amir I. Mohamed

**Affiliations:** 1 Department of Pharmaceutics, Faculty of Pharmacy, King Abdulaziz University, Jeddah 21589, Saudi Arabia; nalhakamy@kau.edu.sa (N.A.A.); oaahmed@kau.edu.sa (O.A.A.A.); aldosarih@gmail.com (H.M.A.); 2 Center of Excellence for Drug Research and Pharmaceutical Industries, King Abdulaziz University, Jeddah 21589, Saudi Arabia; 3 King Fahd Medical Research Center, King Abdulaziz University, Jeddah 21589, Saudi Arabia; 4 Advanced Drug Delivery Research Group, Faculty of Pharmacy, King Abdulaziz University, Jeddah 21589, Saudi Arabia; 5 Oasi Research Institute—IRCCS, Via Conte Ruggero, 73, 94018 Troina (EN), Italy; forgiuseppecaruso@gmail.com (G.C.); fcaraci@unict.it (F.C.); 6 Department of Drug Sciences, University of Catania, 95125 Catania, Italy; 7 Department of Medical Microbiology and Parasitology, Faculty of Medicine, Princess Al-Jawhara Center of Excellence in Research of Hereditary Disorders, King Abdulaziz University, Jeddah 21589, Saudi Arabia; hasfour@kau.edu.sa; 8 Life Science and Environment Research Institute, King Abdulaziz City for Science and Technology (KACST), P.O. Box 6086, Riyadh 11442, Saudi Arabia; mbakhrbh@kacst.edu.sa (M.A.B.);; 9 Department of Biochemistry, Cancer Metabolism and Epigenetic Unit, Faculty of Science, King Abdulaziz University, Jeddah 21589, Saudi Arabia; whabdulaal@kau.edu.sa; 10 Global Analytical and Pharmaceutical Development, Alexion Pharmaceuticals, New Haven, CT 06510, USA; solomon.z.okbazghi@gmail.com; 11 Department of Pharmacology and Toxicology, Faculty of Pharmacy, King Abdulaziz University, Jeddah 21589, Saudi Arabiabeid@kau.edu.sa (B.G.E.); 12 Department of Biomedical Engineering, University of Memphis, Memphis, TN 38152, USA; mkrakula@memphis.edu; 13 Department of Pharmaceutics and Industrial Pharmacy, Military Medical Academy, Cairo 11435, Egypt; miroami@gmail.com

**Keywords:** chitosan, simvastatin, drug release, microparticles, mucoadhesion

## Abstract

This work aimed at improving the targeting and cytotoxicity of simvastatin (SMV) against colon cancer cells. SMV was encapsulated in chitosan polymers, followed by eudragit S100 microparticles. The release of SMV double coated microparticles was dependent on time and pH. At pH 7.4 maximum release was observed for 6 h. The efficiency of the double coat to target colonic tissues was confirmed using real-time X-ray radiography of iohexol dye. Entrapment efficiency and particle size were used in the characterization of the formula. Cytotoxicity of SMV microparticles against HCT-116 colon cancer cells was significantly improved as compared to raw SMV. Cell cycle analysis by flow cytomeric technique indicated enhanced accumulation of colon cancer cells in the G2/M phase. Additionally, a significantly higher cell fraction was observed in the pre-G phase, which highlighted enhancement of the proapoptotic activity of SMV prepared in the double coat formula. Assessment of annexin V staining was used for confirmation. Cell fraction in early, late and total cell death were significantly elevated. This was accompanied by a significant elevation of cellular caspase 3 activity. In conclusion, SMV-loaded chitosan coated with eudragit S100 formula exhibited improved colon targeting and enhanced cytotoxicity and proapoptotic activity against HCT-116 colon cancer cells.

## 1. Introduction

According to the World Health Organization (WHO), cancer caused around 9.6 million deaths in 2018 and is a leading cause of global fatality [1]. Notably, colorectal cancer occupies second place in the most common causes of cancer death (862000 deaths), and accounts for over 9% of all cancer incidence. Unfortunately, to date, surgical resection represents the most common treatment for colorectal cancer and is often also accompanied by the removal of part of the healthy colon or rectum and nearby lymph nodes [2]. In addition to that, the recent drug formulations proposed for the cure of this specific kind of cancer have displayed several side effects [3]. For this reason, the introduction of drug delivery systems which can increase the concentrations of a selected drug at the vicinity of target cancer cells is urgently needed. Chitosan (CHIT) is a natural alkaline polysaccharide derived from chitin, characterized by its non-toxic, biocompatible, hypoallergenic, antibacterial, and biodegradable properties. Most of the properties that render chitosan a desirable carrier for the preparation of drug-loaded NPs are attributable to the primary amine group, which, among many features, is also responsible for chitosan’s cationic nature, controlled drug release, and adhesion to mucosal surfaces [4,5]. As recently highlighted, chitosan-based microparticles (MPs) formulations can release several anti-tumor agents that increase drug internalization into targeted cells and enhance therapeutic effects paralleled by reduced adverse effects [6,7,8]. The term “Eudragit” covers a wide range of non-biodegradable, non-absorbable, and non-toxic polymethacrylate-based copolymers, including neutral as well as charged (anionic and cationic) copolymers prepared by the polymerization of acrylic and methacrylic acids or their esters. Different pH-dependent targeted formulations can be prepared in order to obtain a selective dissolution. For example, at pH higher than 6 Eudragit L100 dissolves, rendering it suitable for enteric coating, while at pH above 7 Eudragit S100 (ES100) is soluble above pH 7 and may be used to target the colon.

Preclinical trials suggest that statins, hydroxymethylglutaryl-CoA reductase inhibitors, possess pleiotropic anticancer properties in many tumors, such as colorectal cancer, by reducing tumor cell growth and survival [9,10]. In particular, as it has been shown by Cho et al., simvastatin (SMV), one of the most representative members of statins, induced apoptosis in a time and dose-dependent manner in the human colorectal HCT-116 and COLO 205 cell lines. It also caused downregulation of the expression of anti-apoptotic proteins [11]. Additionally, in a mouse model of colon cancer associated with colitis, SMV resulted in a significant reduction of tumor development [11]. The novelty of this work is using chitosan from shrimp shells for targeting and controlling SMV release in order to improve its cytotoxic activity and reduce its dose and side effects.

In the present study, the influence of well-characterized SMV-loaded chitosan microparticles coated with Eudragit S100 (SMV-CHIT-ES100 MPs), as well as of its constituents, on HCT-116 cells cytotoxicity was first investigated. In order to shed more light on the potential role of SMV-CHIT-ES100 MPs in the observed decrease in cell viability in these cells, we determined its influence on cell cycle progression, apoptosis (%), and Caspase 3 cellular content. Human colorectal carcinoma cell line (HCT-116) was selected because it represents a preferred model to study the anti-tumor potential of the prepared formula in colon cancer. Lastly, the in vivo colon targeting ability of SMV-CHIT-ES100 MPs was investigated.

## 2. Results

### 2.1. Preparation and Characterization of the SMV-CHIT-ES100 MPs

**Figure 1:**
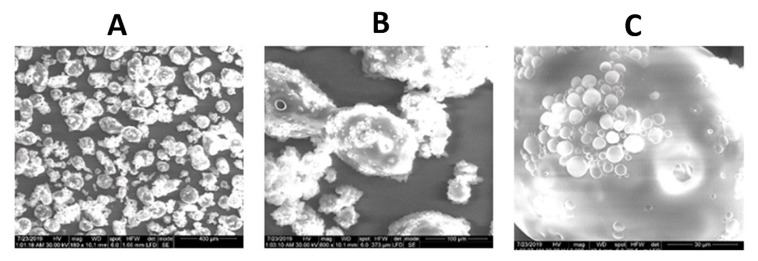
SEM images of characterized simvastatin (SMV)-loaded chitosan microparticles coated with Eudragit S100 (SMV-CHIT-ES MPs) at various magnification powers: 180× (**A**) 800× (**B**) and 3000× (**C**) showing SMV-CH MCPs entrapped within a CHIT-ES100 coat.

SMV was formulated in chitosan MPs in the present study. Prepared MPs were then coated with ES100 to form MPs for colon targeting. SEM images (Figure 1) showed microparticles with an average particle size (172 ± 28 µm), with a smooth surface that showed the entrapment of SMV-Chitosan MPs in the ES100 matrix (Figure 1A–C). 

**Figure 2:**
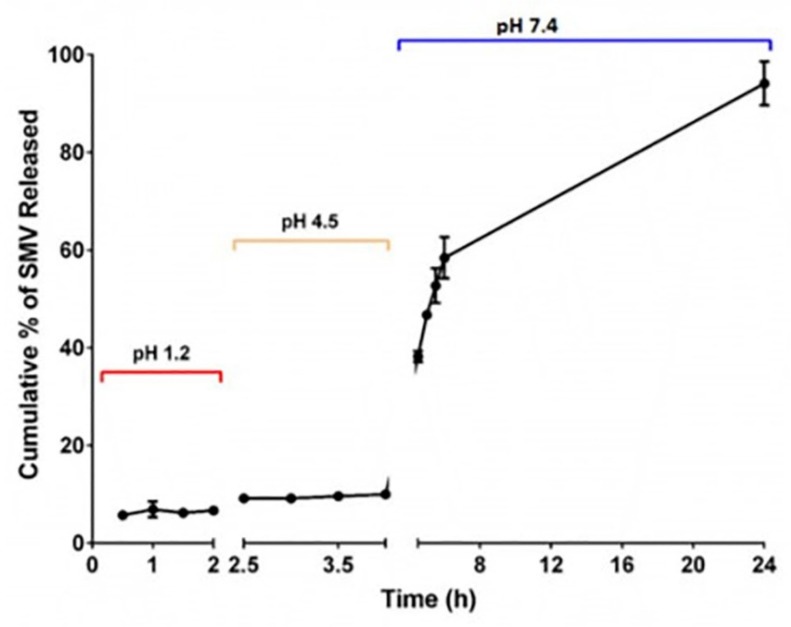
SMV release from SMV-CHIT-ES 100MPs at pH 1.2 for 2 h, pH 4.5 from 2 to 4 h, and pH 7.4 from 4 to 24 h.

SMV-CHIT-ES100 MPs showed EE% of 89.3 ± 4.7. Diffusion assessment aided in further characterization of the formula. Percentage of SMV released from the MPs formula at different pH values (1.2, 4.5 and 7.4) compared with the raw SMV is given in Figure 2. SMV diffused in a pH-dependent manner. SMV released from MPs not exceeded 10% until 4 h (pH values 1.2 and 4.5). After 4 h at pH 7.4, SMV release increased dramatically to reach 100% within 24 h.

### 2.2. In Vitro Antiproliferative Activity

**Figure 3:**
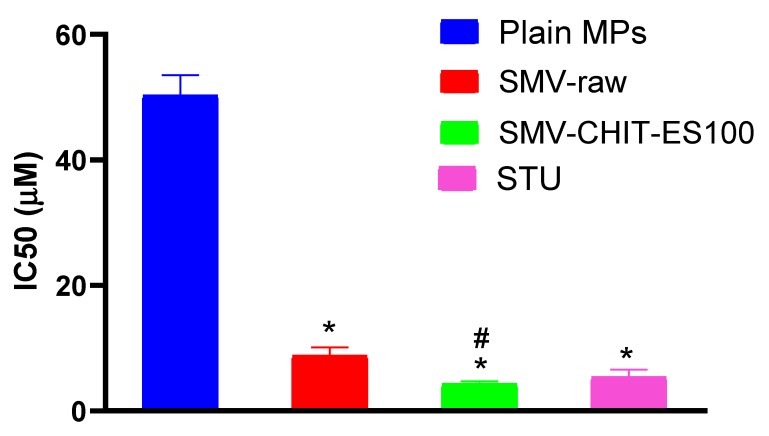
IC_50_ of the raw SMV, Plain MPs and SMV-CH-E S100 MPs in the HCT-116 cell line, as well as Staurosporine (STU). *, Significantly different, (*p* < 0.05) compared to Plain; #, Significantly different (*p* < 0.05) compared to SMV.

The data in Figure 3 indicate that raw SMV exhibited an obviously potent antiproliferative activity against the HCT-116 cells. Plain MPs exhibited weak activity compared to raw SMV with an IC_50_ about 25-fold higher than that of raw SMV. Interestingly, in comparison to raw SMV, encapsulated SMV in CH-E S100 MPs had the greatest proliferation-inhibiting activity (almost double). 

### 2.3. Cell Cycle Progression Analysis

**Figure 4:**
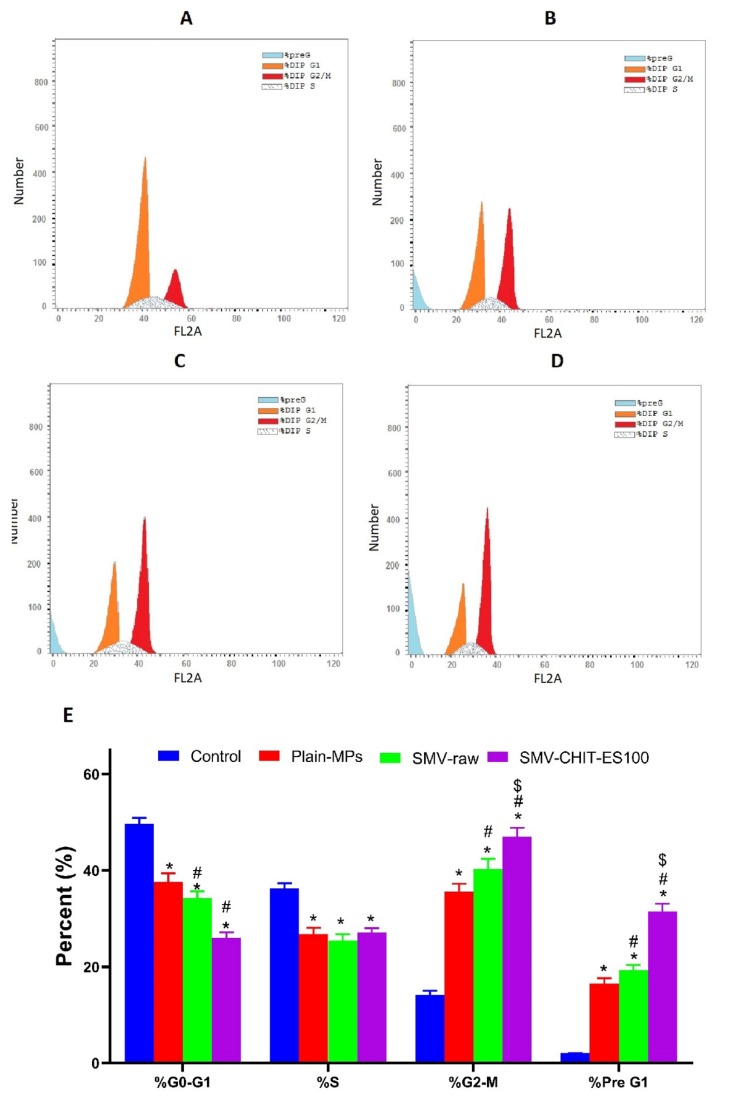
The impact of SMV-CHIT-ES100 MPs on HCT-116 cell cycle phases. (**A**) Control; (**B**) Plain MPs; (**C**) SMV-raw; (**D**) SMV-CHIT-ES100 MPs; (**E**) Graphic representations of each phase. *, Significantly different from control cells at *p* < 0.05; #, Significantly different from Plain MPs at *p* < 0.05; $, Significantly different from raw SMV at *p* < 0.05.

Non-treated HCT-116 cells displayed quick proliferative properties, with the G0/G1 phase giving 49.62 ± 1.8%, the S phase 36.15 ± 2.4%, the G2/M phase 14.14 ± 1.2% and the pre-G1 phase 2.07 ± 0.05% (Figure 4A). Incubation with Plain MPs, SMV and SMV-CHIT-ES100 MPs significantly inhibited the proliferation of the HCT-116 cells and resulted in the accumulation of cells in the G2/M and pre-G phases (Figure 4B–D). In particular, in the pre-G phase the accumulation was 19.28 ± 0.19%, 16.51 ± 0.7% and 31.49 ± 1.02% for Plain MPs, SMV-raw and SMV-CHIT-ES100 MPs, respectively, compared with a control value of 2.07 ± 0.02% (Figure 4E). 

### 2.4. Annexin V–FITC Apoptosis Assay and Caspase 3 Cellular Content

**Figure 5:**
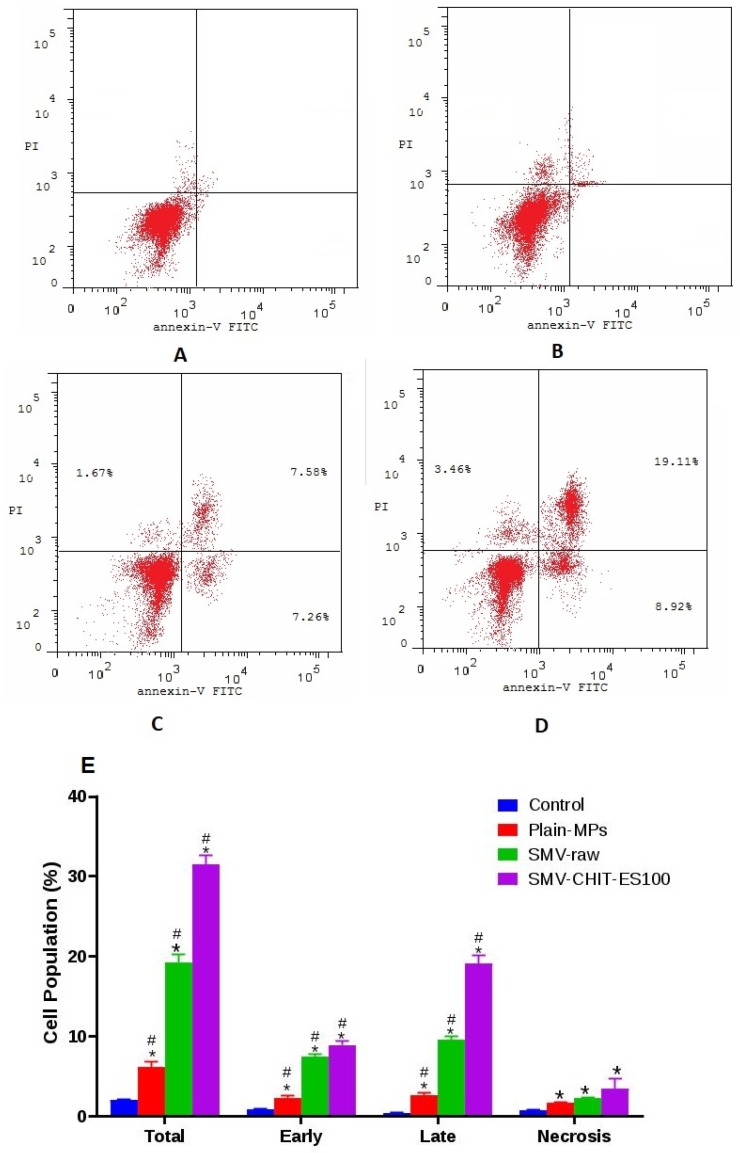
Impact of SMV-CHIT-ES100 MPs on the annexin V FITC positive-staining HCT-116 cells. (**A**) Control; (**B**) Plain MPs; (**C**) SMV-raw; (**D**) SMV-CHIT-ES100 MPs; (**E**) Graphical representations of each phase. *, Significantly different from control cells at *p* < 0.05; #, Significantly different from Plain MPs at *p* < 0.05; $, Significantly different from raw SMV at *p* < 0.05.

The percentage of cells with a positive annexin V stain was assessed in the control, Plain MPS, SMV-raw and SMV-CHIT-ES100 MPs incubations in order to further study the observed cell apoptotic death (Figure 5A–D respectively). In comparison to other incubations, SMV-CHIT-ES100 MPs increased the early, late and total cell death (Figure 5E). 

**Figure 6:**
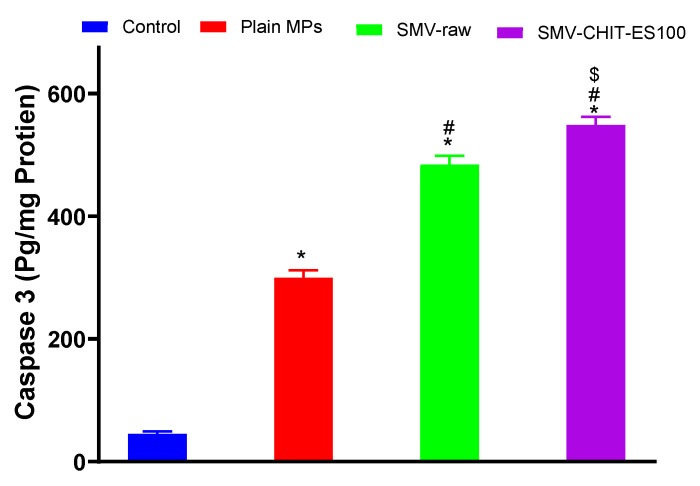
Impact of Plain MPs, SMV-raw, SMV-CHIT-ES100 MPs on caspase 3 enzyme concentrations in HCT-116 cells. *, Significantly different from control cells at *p* < 0.05; #, Significantly different from Plain MPs at *p* < 0.05; $, Significantly different from raw SMV at *p* < 0.05.

The caspase 3 content also confirmed the observed apoptotic cell death induced by the SMV, SMV-CHIT-ES100 MPs. Exposure of the cells to SMV-CHIT-ES100 MPs yielded the highest content of caspase 3 (548.8 ± 13.7 pg/mg protein) when compared to a control value of 45.39 ± 2.62 pg/mg protein and 362.6 ± 1.96 pg/mg protein for raw SMV (Figure 6). 

### 2.5. Realtime X-ray Radiography of Iohexol Formulated in CHIT-ES100 MPs in Rabbits

**Figure 7:**
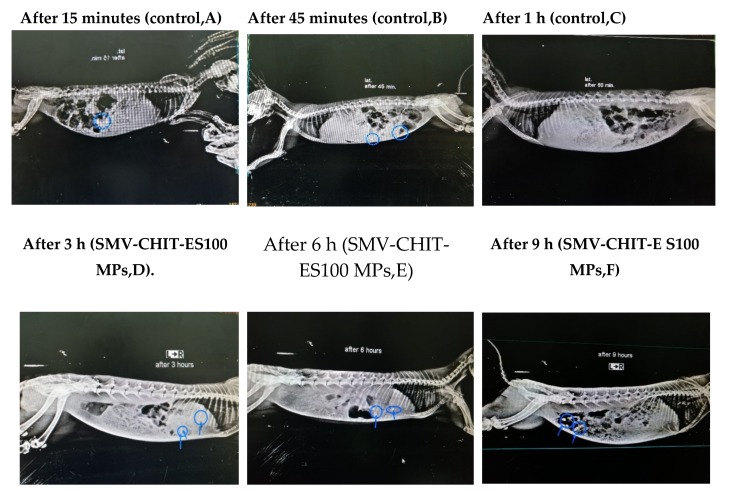
X-ray photographs of (control) after 15 (**A**), 45 (**B**) and 60 min (**C**) and for SMV-CH-E S100 MPs after 3 (**D**), 6 (**E**) and 9 h (**F**).

The data in Figure 7 suggest that iohexol loaded in hard gelatin capsules was detected in the stomach at 15 min and reached the colon at 45 min after oral administration. However, the contrast medium was not detected at 1 h. Thus, no further assessment of the medium in the hard gelatin capsules was performed after 1 h of its administration. On the other hand, iohexol-loaded CHIT-ES100 MPs was detected in the stomach at 3 h and 6 h. After that, the contrast medium was obviously detected in the colon at the period 6–9 h after ingestion. The contrast started to disappear from 9 h and continued thereafter. 

## 3. Discussion

Glutaraldehyde cross-linking was developed for stable SMV-CHIT-ES100 MPs of spherical shape and size of approximately 172 ± 28 µm. The drug entrapment is the result of ionic interaction and hydrogen bonding between CH, glutaraldehyde and SMV, which can be explained well with the high EE percentage of SMV [12,13]. An explanation of the in vitro drug release pattern may be given as such: from 0 to 4 h only less than 10% of SMV was released, which is rationalized by ES100 not dissolving in acidic pH. While SMV release started after 6 h, as the ES100 begins to dissolve at alkaline pH that allows SMV-Chitosan particles to dissolve and dissolute their content. In addition, the degradation of SMV-CHIT-ES100 MPs is a hydrolytic reaction whereby glucosamine-glucosamine, glucosamine-N-acetyl glucosamine and N-acetyl glucosamine-N-acetyl glucosamine links are broken, causing increased release [14,15,16]. Our data indicated that both SMV-raw and SMV-CHIT-ES100 possess potent antiproliferative activity and, in this regard, SMV-CHIT-ES100 showed significant potentiation of SMV cytotoxicity against HCT-116 cells. In general, statins were shown to inhibit the mevalonate pathway [9]. This causes the depletion of the intermediates in the mevalonate pathway, namely farnesyl pyrophosphate (FPP) and geranylgeranyl pyrophosphate (GGPP) [17,18,19]. The depletion of FPP and GGPP causes the disruption of Rho protein prenylation which may explain the cytotoxic effects [20]. In this context, our data are consistent with previous studies reporting the cytotoxicity of SMV. These include SMV cytotoxicity against U266 myeloma cells [3,11], T47D, SKBR3 and MCF-7 breast cancer cells [21,22,23], PC-3 prostate cells [24] and GMK green monkey kidney cells [24].

Analysis of cell cycle phases indicated that colon cancer cells challenged with SMV-raw and SMV-CHIT-ES100 were significantly accumulated in G2/M and pre-G phases, highlighting the proapoptotic activity of SMV. This result is in line with previous reports showing the capability of SMV to cause apoptosis in LipPD1 lipoma cells [25], AXT osteosarcoma cells [26] SK-N-AS neuroblastoma cells [27], PC3 and DU 145 prostate cancer cells [25], D283 and D341 medulloblastoma brain tumor cells [25,26,27,28]. The proapoptotic activity of SMV and SMV-CHIT-ES100 was further confirmed using annexin V staining technique and assessing cellular content of caspase 3 content. Our results revealed significant enhancement of early and late apoptosis as well as caspase 3 content in HCT-116 colon cancer cells. Previous work in C57/BL6 has also shown that SMV is capable of reducing the growth of tumors in the colitis-associated colon cancer (CAC) model. In addition, in order to establish a xenograft model, 53106 COLO205 cells were inoculated subcutaneously into BALB / c nu / nu mice and it was found that SMV-treated animals had smaller tumors, greater areas of necrosis, lower expression of VEGF and higher scores of apoptosis when compared to controls [29,30,31,32]. The mucoadhesive properties of Chitosan and its cationic derivatives are recognized and have been shown to enhance drug adsorption particularly at neutral pH [33,34,35]. Other hypotheses suggested was that Chitosan is a charged polysaccharide capable of adsorbing to a cancer cell. Electrostatic interactions between cancer cells and polycation polymers greatly change the permeability of cancer cells. Tumor cells have not been documented to be damaged directly by Chitosan. Such small oligosaccharides are inhibited by the activation of AMP-activated protein kinase (AMPK) and the mechanistic target of rapamycin (mTOR) [36,37]. Other work has revealed the ability of Chitosan as an immunostimulatory agent which may be used in immunomodulation-related anticancer therapy. Chitosan and its derivatives have been used as an anticancer drug carrier. It has been investigated whether anticancer agents conjugated with chitosan may have anticancer effects with a decrease in side effects and a progressive release of free drugs in cancer tissues [38]. Liposome-chitosan nanoparticles have been used to achieve a dose-dependent tumor-weight drug release inhibition device, which has shown promising results in in vivo studies [39].

Finally, the colon targeting efficiency of the prepared CHIT-ES 100 coat was evaluated by real time X-ray radiography using an iohexol contrast medium in rabbits. Compared to regular hard gelatin capsules, the prepared formula delivered its content into the colon at 6–9 h which is typically the time required for GIT contents to reach the colon [40,41]. These data gain support from our previous observation of SMV release which peaked at a pH of 7.4 in 6–9 h after formula administration. In conclusion, SMV-loaded chitosan coated with eudragit S100 formula displayed enhanced cytotoxicity and proapoptotic activity against HCT-116 colon cancer cells, as well as improved colon targeting efficiency.

## 4. Materials and Methods 

### 4.1. Materials

Eudragit^®^ S100 was gifted by Evonik Industries AG (Essen, Germany). SMV was a gift from SAJA pharmaceuticals (Jeddah, Saudi Arabia). 96-well plates and 12-well plates (Corning transwell^®^ polycarbonate membrane) were obtained from Corning Co., Ltd., (Corning, NY, USA). Chitosan from shrimp shells, Dulbecco’s modified Eagle medium (DMEM), PBS, and the fetal bovine plasma (FBS) and trypsin-0.02% EDTA, were purchased from Sigma Aldrich (St. Louis, MO, USA). Cell counting Kit-8 (CCK-8) was obtained from Boster Biological Technology Co., Ltd., (Wuhan, China). Radio-Immunoprecipitation Assay (RIPA) cell lysates from Vazyme Biotech Co., Ltd., (Nanjing, China) were used. Pentobarbital sodium salt was from Merck Co., Ltd. (Darmstadt, Germany). 

### 4.2. SMV-CHIT-ES100 MPs Preparation

The two-step procedure developed by Thakral et al. was used for the preparation of chitosan microparticles [42]. A 0.5% acetic acid aqueous solution was used briefly to dissolve chitosan into a solution by stirring at ambient temperature. 0.01 mol/L NaOH was used to adjust the pH to 5.5. SMV was dispersed in chitosan solution and the dispersion was passed through 24-gauge needle into liquid paraffin contains 2% span 80 that was stirred at 2000 rpm by a mechanical stirrer. After that, glutaraldehyde (10 mL, 25% v/v) was added to the dispersion. The addition of 5 mL of glutaraldehyde solution followed glutaraldehyde addition by 1 and 2 h. After 1 h, liquid paraffin was decanted, and the collection, washing with petroleum ether and lyophilization of SMV-chitosan microparticles were performed. For encapsulation of core SMV-chitosan microparticles with ES100, an ES100 solution (10% w/v) in acetone-ethanol (2:1) was used for the dispersion of the SMV-chitosan microparticles. This was followed by dropping into mechanically stirred liquid paraffin having 2% v/v span 80. Stirring continued for 4 h. Filtration, washing with petroleum ether and drying of the formed SMV-chitosan coated ES100 microparticles was then carried out.

### 4.3. Characterization of SMV-CHIT-ES100 MPs 

#### 4.3.1. Scanning Electron Microscopy

Double-sided adhesive tape was used to attach the samples to metal stubs to allow for examination. The tape was previously soldered to the stubs and a vacuum was used to provide a gold coating. In order to ensure the SMV-CHIT-ES100 MPs formula surface morphology, a scanning electron microscope (JEM 100-CX; JEOL, Tokyo, Japan) was employed. 

#### 4.3.2. Encapsulation Efficiency 

High-performance liquid chromatography (HPLC)was used after dissolution of the sample in ethanol and filtration through a 0.22 µm filter [43]. Equation (1) was used to calculate the encapsulation efficiency percentage (EE%) of SMV.

(1) $$\mathrm{EE}\;\%=\frac{\mathrm{Amount}\;\mathrm{of}\;\mathrm{SMV}\;\mathrm{in}\;\mathrm{the}\;\mathrm{formula}}{\mathrm{Amount}\;\mathrm{of}\;\mathrm{SMV}\;\mathrm{initially}\;\mathrm{added}}\;\times \;100$$

#### 4.3.3. SMV In Vitro Release from SMV-CHIT-ES100 MPs

Coated microspheres were accurately weighed to correspond to 2 mg of SMV and were placed in a buffer (250 mL, pH 1.2) and magnetically stirred at 50 rpm for 2 h. Centrifugation was carried out and a 0.45 µm membrane filter was used to filter the supernatant for analysis of SMV content. After the 2 h in the buffer (pH 1.2), solution was substituted with 250 mL of buffer pH 4.5 for 2 h and then substituted with 250 mL of buffer pH 7.4. Centrifugation, filtration and analysis of SMV content was carried out for the withdrawn samples. Triplicates of the tests were carried out. 

#### 4.3.4. Cell Culture 

DMEM culture medium, with glutamine and 10% fetal bovine serum was used to raise the stock culture of HCT-116 cells. The medium was replaced every 2 days. Trypsin (2.5%)-EDTA solution (Gibco, Waltham, MA, USA) was used to detach the cells. They were then seeded into sterile 96-well plates. The cell density of each well was 30,000–50,000. Incubation of the cells was performed at 37 °C with 5% CO_2_ content.

#### 4.3.5. Anti-Proliferative Activity 

The cytotoxicity of plain MPs, SMV, SMV-CHIT-ES100 MPs and staurosporine (STU, as a positive control) was determined using MTT assay kit (Sigma-Aldrich, St. Louis, MO, USA) after an incubation period of 48 h with different concentrations of the test compounds at logarithmic intervals. The assay was carried out according to the recommendations of the supplier with minor modifications. The assay was performed in 24-well plates using a volume of 200 µL and a final MTT concentration of 100 µg/mL in the culture medium. After 1.5 h, the medium containing MTT was aspirated off and replaced by the solubilization solution (10% sodium dodecyl sulfate in 0.01 M HCl). The incubation lasted for 2 h. Then, the colored solution was transferred to 96-well plates and measured in an ELISA reader at 540 nm.

#### 4.3.6. Analysis of Cell Cycle Progression

A flowcytometer (FACSCalibur, BD Bioscience) was utilized in the determination of the cell cycle DNA distribution to, as previously described [44]. Briefly, 3 × 105 cells/well were used in seeding the six-well cell culture plates. SMV-CHIT-ES100 MPs equivalent to (0.1 µM) SMV was applied to the cells, and equivalent concentrations of SMV and plain MPs for one day. CycleTEST™ PLUS DNA Reagent Kit (Becton Dickinson Immunocytometry Systems, San Jose, CA, USA) was employed to analyze the cell cycle. The DNA Index (DI) of the tested preparations was determined in reference to cells with a predetermined content of DNA (PBMCs). Staining was carried out using propidium iodide. Finally, CELLQUEST software (Becton Dickinson Immunocytometry Systems, San Jose, CA, USA) was used along with a DNA cytometer to assess cell distribution in the cell cycle phases.

#### 4.3.7. Annexin V Assay

The assay was carried out using the Annexin V-FITC Apoptosis Detection Kit (BioVisionResearch Products, Mountain View, CA, USA). In order to identify cells in early and late apoptosis, Annexin V stain was used along with propidium iodide (PI). PI enters damaged and dead cells, and does not permeate cells with intact membranes. A negative PI and annexin V result suggests that the cells are viable, whereas a positive annexin V and negative PI results suggests early apoptosis. If a positive annexin V and PI result is obtained the cells are considered to be dead or in late apoptosis [17]. 

#### 4.3.8. Caspase-3 Enzyme Assay

HCT-116 cells were cultured in a 96-well plate with 1.8 × 10,000 cells/well for 24 h. Then, cells were exposed to SMV-CHIT-ES100 MPs containing (0.1 µm) SMV or equivalent concentrations of SMV-raw and plain MPs for 24 h. Cells were lysed by a cell extraction buffer and a 100 µL sample from each incubation was used for assaying caspase-3 using a commercial kit (USCN Life Science Inc., Wuhan, Hubei, China).

#### 4.3.9. Realtime X-ray Radiography of the Contrast Medium Iohexol Formulated in Chitosan-Coated Eudrgit in Rabbits 

The handling of rabbits was approved by the Institutional Review Board for Animal Research/Studies who ensured that the care and use of animals conformed to the EU Directive 2010/63/EU on the protection of animals used for scientific purposes. The animals were kept at a temperature of 25 °C in a controlled environment (relative humidity 45%) with light/day cycles. Optimum X-ray radiographic conditions were established (Supplementary file 1). The animals were kept on standard food pellets and had free access to water. Overnight fasted male New Zealand rabbits, weighing 2–2.2 kg were used. The animals were divided into two groups (n = 6). The first group served as a control in which each rabbit was given a single oral plain capsule containing 100 mg of 48.5% w/w iohexol. Animals in the second group were administered capsules containing 100 mg of 48.5% w/w iohexol in chitosan-coated eudrgit prepared as MPs. To prevent capsular destruction, the capsules were kept behind the tongue. The dose of iohexol was chosen based on a preliminary experiment. The animals were kept in rabbit restrainers. The targeting efficiency of the chitosan-coated eudrgit was determined by assessing the contrast generated by iohexol. Radiography was performed at 15, 45 min then after 1, 3, 6 and 9 h, using villa X-ray medical system (Visitor-Italy). 

#### 4.3.10. Statistical Analysis 

Data are presented as the Mean ± SD. Statistical tests were carried out using IBM SPSS^®^ statistics software, version 25 (SPSS Inc., Chicago, IL, USA). Analysis of Variance (ANOVA) followed by Tukey as a post hoc test was used to compare means. *p* < 0.05 indicated statistical significance.

## 5. Conclusions

SMV-CHIT-ES100 MPs were successfully prepared with advantageous properties significant release at pH 7.4 and muco-adhesion to the colonic tissues. The formulated SMV-loaded chitosan and coated with eudragit S100 displayed enhanced cytotoxicity and proapoptotic activity against HCT-116 colon cancer cells, as well as improved colon targeting efficiency.

## Supplementary Materials

The following are available online at https://www.mdpi.com/1660-3397/18/4/226/s1, details of X-ray Dose determination.
