# Supplementary material for: Chitosan Coated Microparticles Enhance Simvastatin Colon Targeting and Pro-Apoptotic Activity
Source: Mar Drugs. 2020 Apr 24;18(4):226. doi: 10.3390/md18040226 (PMC7231066; doi:10.3390/md18040226)
Supplement: Supplementary file 1 [file marinedrugs-18-00226-s001.pdf]

X-Ray Dose Determination

Using villa X-Ray medical system (visitor-Italy), in-vitro X-Ray dose was determined in a pilot experiment. A dose of 100 mg free microspheres was placed in empty hard gelatin capsule size two as a blank group, while 100 mg of contrast media loaded microparticles (48.5% w/w Iohexol/Eudrgit) was placed in capsules and given to the test group. Iohxol chitosam coated Eidragit S100 was prepared as the same procedure for SMV mentioned above except iohexol was added to chitosan solution before dropping in TPP solution. In two 5 cm-diameter glass petri dish capsules of blank group and capsules of test group were exposed to elevated X-Ray doses until complete disappearance in the blank group.
